# Success of intraoperative imaging and management of suspected choledocholithiasis without pre-operative bile duct imaging – A case series

**DOI:** 10.1016/j.amsu.2018.10.036

**Published:** 2018-11-05

**Authors:** Timothy Platt, Kristy Smith, Martha Nixon, Surajit Sinha, Gandrapu Srinivas, Stuart Andrews

**Affiliations:** a General Surgery, South Devon NHS Foundation Trust, United Kingdom; b Core Surgical Trainee, Norfolk and Norwich University Hospitals NHS Foundation Trust, United Kingdom; c StR General Surgery, Plymouth Hospitals NHS Trust, United Kingdom; d Locum Consultant Upper GI Surgery, South Devon NHS Foundation Trust, United Kingdom; e Consultant Upper GI Surgeon, South Devon NHS Foundation Trust, United Kingdom

**Keywords:** Choledocholithiasis, Bile ducts, Extrahepatic, Cholecystectomy, Laparoscopic, Biliary tract surgical procedures, Gallstones

## Abstract

**Background:**

Laparoscopic common bile duct exploration (LCBDE) is gaining popularity over endoscopic retrograde cholangiopancreatography (ERCP) for the management of common bile duct stones. However, its application has been almost exclusively following preoperative stone confirmation via magnetic retrograde cholangiopancreatography (MRCP), endoscopic ultrasound (EUS) or ERCP. We present our series of LCBDE following detection of common bile duct stones with intraoperative imaging (IOI) alone, in consecutive elective and emergency patients with suspected choledocholithiasis.

**Materials and methods:**

All patients with suspected but unconfirmed choledocholithiasis undergoing LC with intention to proceed to LCBDE between January 2015 and June 2017 were included. LCBDE was performed following the discovery of choledocholithiasis on IOI.

**Results:**

371 patients with suspected choledocholithiasis underwent LC with IOI. CBD stones or obstructing sludge was identified in 107 patients (29%), with sensitivity of 96.2% and specificity of 98.5%. 100 patients, median age 59, went on to have LCBDE as indicated by intraoperative imaging. 76% were performed as emergency cases and conversion to open rate was 2%. There were no mortalities. Bile leak and retained stones occurred in 4% and 3% respectively. 7/100 patients required re-intervention, with re-look laparoscopy (n = 4) and ERCP (n = 3). Median length of stay was 1.5 and 3 days for elective and emergency cases respectively, and 30 readmission rate was 8%.

**Discussion and conclusion:**

Traditionally patients presenting with suspicion of choledocholithiasis undergo preoperative MRCP/EUS and/or ERCP prior to eventual LC. We propose an alternative, more streamlined, pathway of treatment without requiring preoperative cholangiography, applicable to both elective and emergency patients.

## Highlights

• Laparoscopic common bile duct exploration is a safe and effective treatment for bile duct stones.
• Intraoperative cholangiography is sensitive and specific in identification of bile duct stones.
• Bile duct exploration can be safely performed on the basis on intraoperative imaging alone.
• Intraoperative imaging provides sufficient information for real time decision making.

## 1 Introduction

Gallstones are common and present in around 15% of the UK adult population [1]. Approximately 1 in 5 individuals with asymptomatic gallstones will go on to develop complications over a 10 year period [2]. The incidence of common bile duct (CBD)stones in patients presenting with gallstone disease lies at approximately 10–15% [[3], [4], [5]].

The initial investigations in patients presenting with symptoms of gallstone disease often includes biochemical markers and abdominal ultrasound scan (US), which have low diagnostic accuracy in identifying patients with CBD stones [6]. Depending on local resources many patients with clinical features suggestive of CBD stones will typically undergo preoperative cholangiography in the form of magnetic resonance cholangiopancreatography (MRCP), endoscopic ultrasound (EUS) or endoscopic retrograde cholangiopancreatography (ERCP). Alternatively patients can undergo an intraoperative imaging (IOI) in the form of cholangiogram or ultrasound scan of the bile duct. Patients with CBD stones can be treated with a two stage approach consisting of ERCP and laparoscopic cholecystectomy or a single stage laparoscopic cholecystectomy (LC) and laparoscopic common bile duct exploration (LCBDE). The latter has become established as a safe alternative to ERCP [[7], [8], [9]]. When comparing cost effectiveness, length of stay and return to work, LCBDE has been shown to have advantages [7,10,11]. Despite this, its application has been almost exclusively used in highly selected elective patients where a CBD stone has been confirmed pre-operatively [[12], [13], [14]]. Despite the prevalence of CBD stones, LCBDE is therefore a relatively infrequently performed procedure relative to the disease burden.

In this study, we present outcomes of LCBDE in patients in a medium sized district general hospital performing a high volume of LCBDE cases, using only intra-operative imaging for CBD stone confirmation.

## 2 Methods

All patients with gallstone disease and suspected, but unconfirmed, choledocholithiasis undergoing LC under the care of 2 surgeons (GS & SA) at a district general hospital between January 2015 and June 2017 were included. Both surgeons had experience of LCBDE (>50 cases each) prior to the start of the study period. Choledocholithiasis was suspected on the basis of elevated liver function tests (LFTs) or a dilated bile duct on abdominal US, which was defined as >5 mm+1 mm for every decade of life over 50 years. These patients were treated with LC with intention to proceed to LCBDE when feasible and indicated by IOI. Patients subsequently undergoing LCBDE on the basis of IOI were analysed. Data pertaining to these patients was extracted from a prospective database of all LCBDEs performed in our unit, including demographics, operative details, pre- and post-operative imaging, admissions, complications and follow-up. Comparison with electronic patient records was performed for accuracy.

This study was registered online (www.researchregistry.com, UIN: researchregistry4262) and reported in compliance with the PROCESS guidelines [15].

In our unit, patients presenting with suspected gallstone disease are assessed with a clinical history and examination, LFTs and abdominal US. Some patients, particularly the elderly, may then undergo computed tomography (CT) scan to rule out an alternative pathology such as pancreatic or liver cancer. Following fluid resuscitation and optimization where appropriate, all patients are then counselled and considered for laparoscopic cholecystectomy with intra-operative bile duct imaging via laparoscopic US (LUS) or on-table cholangiogram (OTC). The surgeon will then go on to perform laparoscopic CBDE when CBD stones are identified. In our unit MRCP or ERCP are not routinely used prior to laparoscopic cholecystectomy for patients with suspected bile duct stones.

LCBDE can be performed via either a trans-cystic (TC) approach or via choledochotomy (CD). The suitable approach is decided based on various factors including caliber and length of cystic duct, CBD diameter, size of CBD stones, and accessibility of CBD (Table 1). Where possible the trans-cystic approach is preferred due to the potential morbidity associated with choledochotomy. Choledochotomy is reserved for those patients unsuitable for a trans-cystic exploration. In patients unsuitable for either approach for reasons of anatomy or tissue fibrosis, laparoscopic cholecystectomy is completed followed by post-operative ERCP.

**Table 1:** Parameters defining suitability for trans-cystic (TC) or trans-ductal (TD) LCBDE.

Trans-cystic LCBDE	Trans-ductal LCBDE
- Non-tortuous cystic duct	- CBD luminal diameter >1 cm
- Cystic duct dilation to allow intubation with 2 mm choledochoscope	- CBD accessibility not ruled out by fibrosis or inflammation
- Small CBD stones able to be removed via cystic duct	

## 3 Results

### 3.1 Demographics

371 patients with gallstones and suspected but unconfirmed choledocholithiasis underwent LC with IOI in our unit between January 2015 and June 2017. Choledocholithiasis or obstructing sludge were identified in 107 patients (28.8%). In 14 patients the CBD was inaccessible or unsuitable for LCBDE, and ERCP was performed postoperatively. The remaining 93 patients proceeded onto LCBDE was on the basis of intraoperative imaging only. An additional 7 patients underwent LCBDE on the basis of a grossly dilated duct and no flow of contrast into the duodenum on OTC, without confirmation of CBD stones. These 7 were all performed via the transcystic route, and no stones were found in this subgroup. Thus, a total of 100 consecutive patients underwent LCBDE on the basis of intraoperative imaging only.

### 3.2 LCBDE cohort

**Fig. 1:**
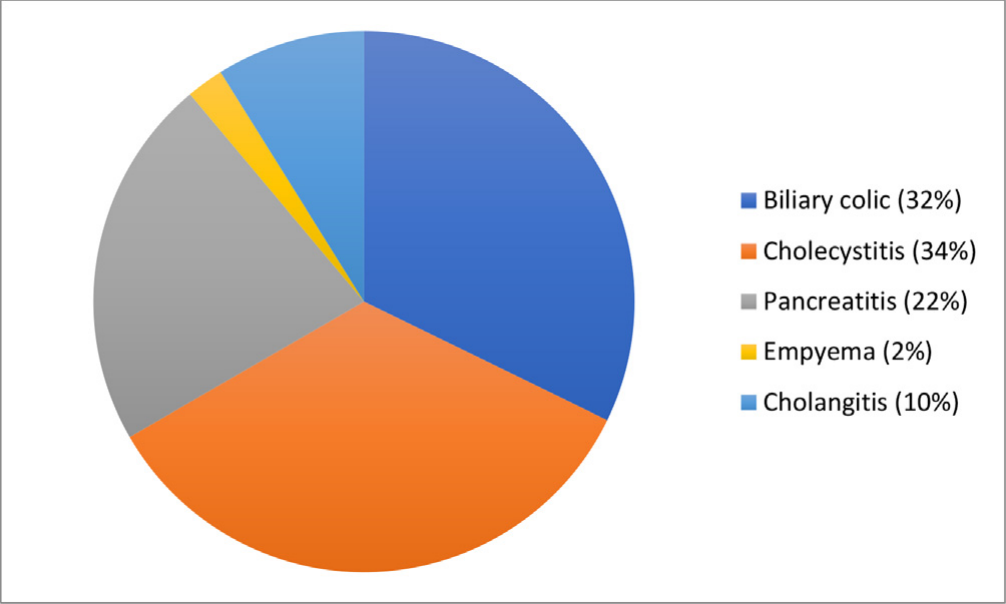
Presenting conditions indicating LC.

Of our cohort undergoing LCBDE, the median age was 59 years (range 17–84 years). The male to female ratio was 1–1.6. ASA grades were as follows: ASA 1: n = 20, ASA 2: n = 66, ASA 3: n = 14. Indications for surgery were biliary colic, acute cholecystitis, gallbladder empyema, cholangitis or pancreatitis, with suspected choledocholithiasis (Fig. 1).

### 3.3 LCBDE operative details

Of 100 LCBDEs, 24 performed electively while 76 were emergency procedures. 98 cases were completed laparoscopically, with a conversion to open rate of 2%. Both these cases were converted due to a large stone impacted in the distal CBD, which was impossible to engage with endoscopic technique. CBDE was performed via a TC route in 45 and via CD in the remaining 55. In these cases, primary CBD closure was performed in 48, and 7 had biliary decompression via T-tube. Indications for T-Tube insertion included severe cholangitis (n = 4), pancreatitis with ampullary oedema and inflammation of the porta (n = 1), immunosuppression therapy (n = 1), and chronic inflammation of CBD due to multiple previous ERCPs (n = 1). 6 of the cases using T-tube were during the first quarter of the cohort while the remaining case was in August 2016, indicating the increasing confidence in primary choledochotomy closure.

Median operating time (OT) was 168 min, and was significantly shorter for LCBDE performed via transcystic route than via choledochotomy (152 vs 195, p = 0.0030). Interestingly, there was no significant difference in OT between elective and emergency cases (171 vs 167 min, p = 1.0). However, comparison of OT between the first 50 and second 50 LCBDEs performed demonstrated a significant decrease in operating time (198 vs 152 min, p < 0.00001).

### 3.4 Length of stay

Median post op length of stay following CBDE was 2 days overall, and was 1.5 and 3 days for elective and emergency cases respectively.

### 3.5 Complications

Patients were observed in this study for a median time of 186 days, with 88 patients having a least 6 weeks of data. No patients died in the 30 days following LCBDE. 8 required readmission to hospital within 30 days for complications related to their procedure or uncontrolled post-operative pain.

Overall complication rate was 18%. Major complications included bleeding (2%), retained stone (3%, including 1 patient who developed pancreatitis), bile leak (4%), and interiorisation of drain (1%). Post operative ERCP for either bile leak or CBD stone retention was performed in 5%. All patients suffering from bile leak or retained stones were in the choledochotomy group, however in comparison with the transcystic group this did not reach significance (p = 0.07). 4% required re-laparoscopy for bile leak (n = 2), bleeding from port site (n = 1) and interiorisation of drain (n = 1).

Minor complications included pneumonia (3%), small pleural effusion (1%), intra-abdominal collection managed conservatively (4%).

Including all recorded complications, there was no significant difference in the complication rates between the elective and emergency groups (p = 0.53).

### 3.6 Diagnostic accuracy of intraoperative imaging

As per the design study, no patients in this study had confirmed CBDE stones prior to surgery. In the 107 patients in whom choledocholithiasis or sludge was identified intraoperatively, stones were found in 100, and obstructing sludge in 3, while no stones were found in 4 patients. Of the 264 patients with negative IOI, 4 presented with retained stones in the postoperative period. Thus the sensitivity of IOI in our hands is 96.2%, with a specificity of 98.5%.

## 4 Discussion

Patients with CBD stones have the potential to develop serious complications such as pain, cholangitis, hepatic abscess, biliary obstruction, secondary biliary cirrhosis and portal hypertension. In view of this, recommendations from the British Society of Gastroenterology [16] and from European Association of Endoscopic Surgeons [17] are that, when possible, they are removed.

A Cochrane review comparing single (LCBDE during LC) vs two-stage (ERCP followed by LC) approaches to bile duct stones demonstrated similar safety and efficacy [7], while others, including individual randomized controlled trials and a meta-analysis have demonstrated lower morbidity, shorter length of stay and improved cost-effectiveness [[18], [19], [20], [21], [22]].

Despite the widespread adoption of laparoscopic cholecystectomy since the early 1990's, and the evidence supporting LCBDE, the employment of laparoscopic common bile duct exploration has been rather more reserved. Even the larger published case series are modest compared to numbers of patients undergoing laparoscopic cholecystectomy [13,18,23,24]. For example, Quaresima et al. have recently published results of 384 patients undergoing LCBDE over a 23 year period [25], while Aawsaj et al. published outcomes of 296 patients over a 15 year period, indicating a practice of only 17 and 20 cases per year respectively [13]. Potential factors underlying this include ease of access to and the long-established safety of ERCP, limited access to theatre time or equipment, technical difficulty of LCBDE, and concerns about the risk of complications from LCBDE. As a result LCBDE is often offered to very selected patients [12] and generally in the elective setting [24,26].

In contrast, 76% of cases in our series were performed in the emergency setting, including patients presenting with acute cholecystitis, acute pancreatitis and cholangitis. Despite this the stone clearance rate was 97%, with conversion to open rate of only 2%. Darrien et al. had a similar incidence of emergency cases in their series of 216 bile duct explorations, but with a stone clearance of 87%, with 32% of cases being converted to open [14]. Our findings, demonstrating the safety and efficacy of LCBDE in the emergency setting is consistent with those of Chan et al. who found similar morbidity, mortality and LOS when comparing LCBDE in the emergency and elective settings [23].

In our experience almost all patients, when given the choice, opt for a single-stage approach, consistent with the findings of others [27]. With this in mind, all patients presenting to our unit with gallstone disease and suspected CBD stones, are routinely offered laparoscopic cholecystectomy with intra-operative imaging, and, where appropriate, progression onto LCBDE when CBD stones are found. MRCP or EUS is not routinely performed prior to LC and ERCP is only performed in patients unfit for surgery, at the expressed wishes of the patient, or when stone clearance is not possible at LCBDE. Consequently, despite being a small/medium sized district general hospital, we perform around 100 LCBDEs per annum.

To our knowledge this is the first series of LCBDE based on intraoperative imaging alone, and does not include any patients in whom choledocholithiasis was confirmed by pre-operative MRCP. While numerous series describe outcomes of LCBDE with IOI, preoperative MRCP is invariably used in these series for stone confirmation [14,24]. Therefore, from these series, it is difficult to establish the safety and efficacy of LCBDE based on IOI alone. Our study, however, included 371 patients, suspected to have choledocholithiasis, in whom IOI was used as an alternative, rather than an addition, to MRCP. In our view, while MRCP has advantages, including CBD stone identification and delineation of anatomy, its routine use in these patients has certain disadvantages. Firstly in elective patients, CBD stones may occur in the interval between MRCP and surgery, thus a negative MRCP may be falsely reassuring. Secondly, in the emergency setting, preoperative MRCP may delay surgery, increasing inpatient stay.

As we demonstrate, intraoperative imaging is highly accurate in identifying choledocholithiasis, provides necessary information for real time decision making regarding both suitability of the bile duct for exploration and appropriate modality of LCBDE. In 45% of cases, CBD clearance was achieved via the transcystic route, while the remaining 55% required choledochotomy. Reinders et al. performed a systematic review demonstrating higher bile leak rate after choledochotomy (11%) compared to transcystic route (1.7%) [28]. In our cohort, all 4 bile leaks were in the choledochotomy group, giving a leak rate post choledochotomy of 7% and post TC-LCBDE of 0%. Reinders et al. suggest that choledochotomy is only performed by experienced surgeons [28]. We agree with that sentiment, though argue that given the high frequency of CBD stones, even in smaller units such as ours, surgeons with subspecialty interest can readily become experienced in LCBDE via both choledochotomy and transcystic routes.

Of 55 patients undergoing choledochotomy, all had primary closure except 7, who had T-tube insertion. Six of these were in the first year of our cohort, reflecting the learning curve and increase in confidence in choledochotomy closure in our unit. Primary choledochotomy closure has been shown to be associated with fewer complications than T-tube insertion in a comprehensive systematic review and meta-analysis [29]. However, most studies included in this meta-analysis excluded patients with cholangitis or pancreatitis, and some consisted of only elective patients. Indeed, in the largest UK cohort study demonstrating the safety of primary choledochotomy closure, only 10% of procedures were emergency [24]. Our series confirms the safety of choledochotomy and primary closure in the emergency setting. However, T-tube drainage should be considered for some patients, including those with severe CBD inflammation due to cholangitis or ampullary oedema/compression from severe pancreatitis.

The main limitation of this study was the passive nature of follow up. Most complications of LCBDE occur prior to discharge. However in order to pick up retained stones we relied on patients representing with symptoms or complications. It is feasible that some may therefore be missed, though we surmise that all clinically significant retained stones are likely to have represented, and only clinically insignificant retained stone have been missed. Furthermore, while this study uses IOI in place of pre-operative MRCP/EUS, we are unable to make a direct comparison of the impact this has on the patient pathway and surgical outcomes. Further research including randomized trials is required to establish this.

In conclusion, based on our study LCBDE is safe and effective for the treatment of choledocholithiasis in both elective and emergency cases, and can be performed on the basis of intraoperative imaging without the need for preoperative cholangiography. IOI is also able to identify key features of biliary anatomy to aid real time decision making regarding the suitability and modality of LCBDE.

## Ethical approvalr

Not required.

## Sources of funding

None.

## Author contribution

T Platt – study design, data collection, analysis, write up.

K Smith – data collection.

M Nixon – Data collection and write up.

S Sinha – data collection.

G Srinivas – study design, manuscript review.

S Andrews – Study design, manuscript review.

## Conflicts of interest

None.

## Research registration number

Researchregistry4262.

## Guarantor

S Andrews.

## Trial registry number

None.

## Provenance and peer review

Not commissioned, externally peer reviewed.

## Appendix A Supplementary data

Annals PROCESS checklist Annals PROCESS checklist
